# Robustness of network attack strategies against node sampling and link errors

**DOI:** 10.1371/journal.pone.0221885

**Published:** 2019-09-04

**Authors:** Momoko Otsuka, Sho Tsugawa

**Affiliations:** Graduate School of Systems and Information Engineering, University of Tsukuba, Tsukuba, Ibaraki, Japan; University of Sao Paulo, BRAZIL

## Abstract

We investigate the effectiveness of network attack strategies when the attacker has only imperfect information about the network. While most existing network attack strategies assume complete knowledge about the network, in reality it is difficult to obtain the complete structure of a large-scale complex network. This paper considers two scenarios in which the available network information is imperfect. In one scenario, the network contains link errors (i.e., missing and false links) due to measurement errors, and in the other scenario the target network is so large that only part of the network structure is available from network sampling. Through extensive simulations, we show that particularly in a network with highly skewed degree distribution, network attack strategies are robust against link errors. Even if the network contains 30% false links and missing links, the strategies are just as effective as when the complete network is available. We also show that the attack strategies are far less effective when the network is obtained from random sampling, whereas the detrimental effects of network sampling on network attack strategies are small when using biased sampling strategies such as breadth-first search, depth-first search, and sample edge counts. Moreover, the effectiveness of network attack strategies is examined in the context of network immunization, and the implications of the results are discussed.

## Introduction

The network attack problem has received much attention, and several network attack strategies have been proposed [1–4]. The network attack problem involves finding a (small) set of nodes whose removal fragments the network [1]. Herein, we refer to such node removal strategies as network attack strategies. The effectiveness of these network attack strategies is evaluated based on the network connectivity after node removal, a popular evaluation measure being the size of the giant component (GC) [5]. Typically, the network attack problem is solved using measures of node influence, based on which node rankings are obtained and then nodes are removed in the order of those rankings. As measures of node influence, centrality measures such as degree and betweenness centrality [6] are widely used [5]. Recently, a scalable measure known as collective influence (CI) has been proposed and has been shown to be effective for the network attack problem [1]. Network attack strategies have various applications, such as viral marketing [1, 7, 8], evaluation of network resilience [9, 10], and network immunization [5, 11, 12].

While most existing network attack strategies assume complete knowledge about the network [1, 2, 4], in reality it is difficult to obtain the complete structure of large-scale complex networks [5, 13–16]. Because real-world networks such as social networks and the Internet are huge, obtaining the entire network structure is prohibitive, and typically only part of the network structure is available from network sampling [17–19]. Even if the target network is not so large, it can still be difficult to obtain its complete structure [5]. For instance, in gene networks the relationships among nodes are inferred from noisy measurements that produce link errors [20]. In social networks, it is fundamentally difficult to observe social relationships such as friendship and trust [21, 22]. If information about the network is imperfect, then the effectiveness of network attack strategies may be degraded.

Melchionna et al. [5] performed pioneering work to evaluate the effectiveness of network attack strategies when the attacker has imperfect information about the network. Performing simulated attacks on synthetic networks, Melchionna et al. introduced link errors (i.e., missing and false links) into the networks and then evaluated the effectiveness of network attack strategies using those networks with link errors. Their results show that particularly for scale-free networks, network attack strategies are robust against link errors.

While the effects of link errors on network attack strategies have been studied, the effects of network sampling, which is a typical cause of errors introduced in the networks, on network attack strategies have not been studied before. The networks of interest nowadays are huge, and network sampling is unavoidable to analyze such large-scale networks [17–19]. However, the answers to the following questions remain unclear: (1) Which attack strategy should we use when only the small fraction of the network structure is available? (2) Which network sampling method should we use for exploring the large-scale network? To answer these questions is important when applying network attack strategies to real large-scale networks.

In this paper, we aim to reveal the effectiveness of network attack strategies when the attacker has only imperfect information about the network, extending the work by Melchionna et al. [5]. We consider two scenarios in which the available network information is imperfect. One scenario is the same as in [5] and assumes that the network contains link errors due to measurement errors. The other scenario, which was not considered in previous studies, assumes that the target network is so large that only part of the network structure is available from network sampling. Consequently, we investigate the effects of both link errors and network sampling on the effectiveness of network attack strategies, and aim to answer the above questions.

The main contributions and findings in this paper can be summarized as follows.

- We evaluate the effectiveness of CI, a state-of-the-art network attack strategy, under imperfect information. Our results show that CI is effective when the complete knowledge on the network is available whereas CI is sensitive against network uncertainty. When the level of network uncertainty is large, the effectiveness of CI is comparable with that of the conventional Degree strategy.
- We investigate how the network sampling affects the effectiveness of network attack strategies. We show that the attack strategies are far less effective when the network is obtained from random sampling, whereas the detrimental effects of network sampling on network attack strategies are small when using biased sampling strategies such as breadth-first search, depth-first search, and sample edge counts.
- We evaluate the effectiveness of network attack strategies in the context of network immunization [23, 24]. Our results suggest that the effects of link errors and sampling are not so severe when the attack strategies are applied to immunization.

## Methodology

We investigated the connectivity of the undirected network *G* = (*V*, *E*) when a proportion *p* of all nodes was removed based on the attack strategy. For this study, we assumed that perfect information about network *G* was unavailable and the attackers only know network *G*′ = (*V*′, *E*′), a network with unknown errors. We used the attack strategies to determine which nodes of network *G*′ to attack. We then investigated the connectivity of the true network *G* with the same nodes removed.

**Fig 1:**
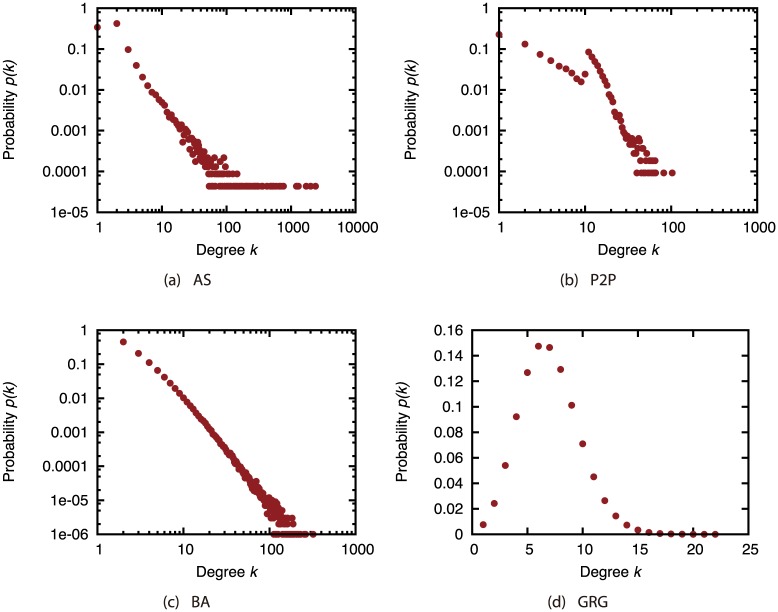
Degree distributions of the target networks.

We used two types of real network for network *G*. One is a network that expresses connections between autonomous systems (AS) (available at http://www-personal.umich.edu/~mejn/netdata/), and the other is a network that expresses connections between peers, namely a peer-to-peer (P2P) network [25, 26] (available at http://snap.stanford.edu/data/p2p-Gnutella04.html). We also used two types of synthetic network for network *G*. For generating synthetic networks, we used the Barabási–Albert (BA) model [27] and the Geometric Random Graph (GRG) model. The BA model generates networks with power-law degree distribution. The GRG model generates random networks where the connection between nodes is determined based on the geographical coordinates of the nodes. Using the BA and GRG models, we generated 100 networks where the number of nodes is 10,000. For the parameter of the BA model, we used *m* = 2 for generating networks with similar average degree with the AS network. For the GRG model, the radius for connecting nodes was 0.015 to generate connected networks while making the average degree be not far from other networks. BA graphs and GRG graphs were generated with R igraph package (https://igraph.org/). The characteristics of each network are shown in Table 1, and Fig 1 shows the degree distributions of each network. For BA and GRG networks, the values shown in Table 1 are averaged for the 100 generated networks. According to Fig 1, the GRG network has normal-like degree distribution while other networks have power-law-like degree distributions. We can also find that the AS network has nodes with extremely large degrees compared with the P2P and BA networks. Namely, the AS network has highly skewed degree distribution compared with other networks.

**Table 1:** Statistics of target networks.

	AS	P2P	BA	GRG
No. of nodes	22,963	10,876	10,000	10,000
No. of links	48,436	39,994	19,997	34,874.3
Average degree	4.218	7.354	3.999	6.975
Clustering coefficient [28]	0.0111	0.00540	0.00210	0.591
Average shortest path length	3.842	4.635	5.393	51.671

We created two incomplete networks *G*′ from network *G*: a network with link errors and a sampled network. We created the network with link errors using the same procedure as in Melchionna et al. [5]. Specifically, we removed |*E*|*δ* (0 ≤ *δ* ≤ 1) randomly selected links from network *G* and added |*E*|*α* (0 ≤ *α* ≤ 1) links to randomly selected node pairs within the network to create incomplete network *G*′. Here, *α* and *δ* are the parameters that controlled the magnitude of the incompleteness. To create the sampled network, we followed the procedure outlined by Tsugawa and Kimura [13]. Specifically, we used four different methods of sampling, namely (i) breadth-first search (BFS), (ii) depth-first search (DFS), (iii) sample edge count (SEC) [17], and (iv) random sampling (RAND), to sample the proportion *p*_*s*_ of nodes from network *G* to create incomplete network *G*′. Sampling node *v* enabled us to obtain the associated links. In incomplete network *G*′ = (*V*′, *E*′), *V*′ is the set of sampled nodes, and *E*′ is the set of links between the set of nodes in *V*′.

Using incomplete network *G*′, we determined which nodes to remove based on three different attack strategies, namely (i) a strategy based on degree centrality (Degree) [1], (ii) a high degree adaptive (HDA) strategy based on recalculations of degrees [1], and (iii) a strategy based on CI [1]. We removed the same nodes from network *G* and then evaluated its connectivity by using the size of its GC as the evaluation index.

Furthermore, in this study we simulated the spread of viruses to evaluate the effectiveness of attack strategies when applied to the immunization problem. We used incomplete network *G*′ to determine which nodes to attack. We immunized the nodes to be attacked in network *G* and simulated the virus diffusion based on the susceptible–infected–removed (SIR) model [29]. The SIR model comprises three states, namely susceptible (S), infected (I), and removed (R). However, immunized (M) nodes are not involved in virus spread. At time *t* = 0, we randomly selected one non-immunized node in network *G* and changed its state to infected. The remainder of the non-immunized nodes are susceptible (but not infected). At any given time *t*, infected nodes *v* cause adjacent susceptible nodes *u* to become infected at infection rate *β*. In addition, infected nodes transition to the removed state at recovery rate *γ*. To serve as an index to quantify the extent of virus spread, we determined the number of non-infected nodes (susceptible and immunized nodes) at the time when the aforementioned stochastic processes converge and node states stop changing. The stochastic processes were realized using the random number generator in the Python NumPy module (https://www.numpy.org/).

For the ensuing experiments, we created 100 incomplete networks *G*′ for each parameter used to determine the magnitude of incompleteness. For each incomplete network *G*′, we determined the nodes to attack, and calculated the size of the GC, and the average of the GCs were obtained. In addition, we conducted 1,000 independent virus spread simulation runs for each incomplete network *G*′ to determine the average number of non-infected nodes. We used *l* = 1 and *l* = 2 as parameters for the CI attack strategy. In addition, we used an infection rate of *β* = 0.1 and a recovery rate of *γ* = 0.01 in the virus spread simulations.

## Results and discussion

### Effects of link errors

**Fig 2:**
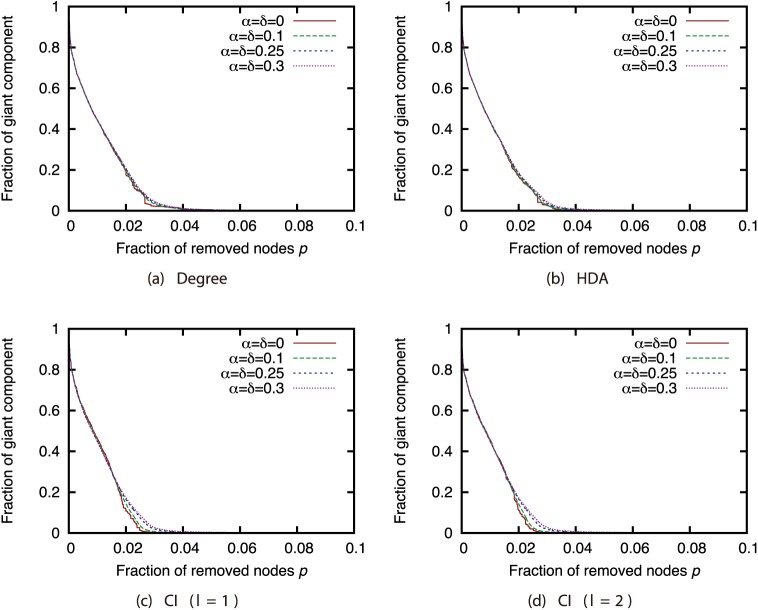
Fraction of GC vs. fraction *p* of removed nodes: Comparison of magnitude of incompleteness (network: AS).

**Fig 3:**
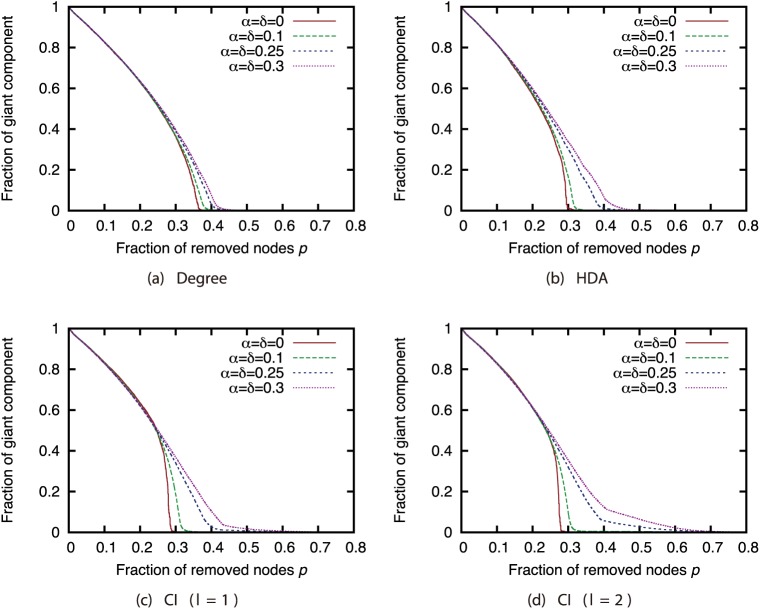
Fraction of GC vs. fraction *p* of removed nodes: Comparison of magnitude of incompleteness (network: P2P).

**Fig 4:**
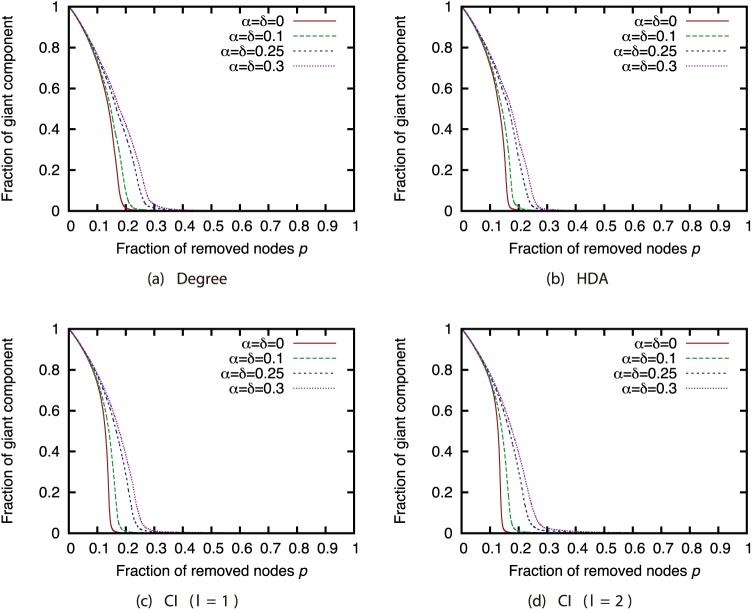
Fraction of GC vs. fraction *p* of removed nodes: Comparison of magnitude of incompleteness (network: BA).

**Fig 5:**
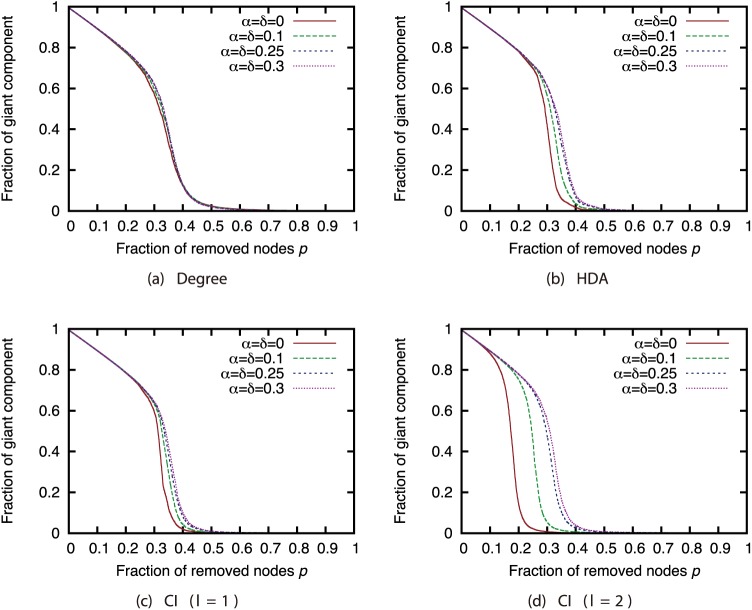
Fraction of GC vs. fraction *p* of removed nodes: Comparison of magnitude of incompleteness (network: GRG).

First, we investigated how link errors affected the effectiveness of each attack strategy. Figs 2, 3, 4 and 5 show the relationship between the proportion *p* of nodes removed from the AS, P2P, BA, and GRG networks and the proportion of the largest connected components when the nodes are removed. These figures compare different magnitudes of incompleteness.

In the AS network, the magnitude of incompleteness has hardly any effect on the size of the largest connected components under each of the attack strategies (Fig 2). In contrast, in other networks, the effectiveness of the attack strategies declines as the incompleteness of link information increases. In particular, the effects are substantial under the HDA and CI strategies. Previous study [5] indicates that link errors have an extremely minor effect on attack strategies in networks with heavily skewed degree distribution. The degree distribution is more skewed in the AS network than in the other networks (Fig 1); thus, our results are consistent with those of previous research.

**Fig 6:**
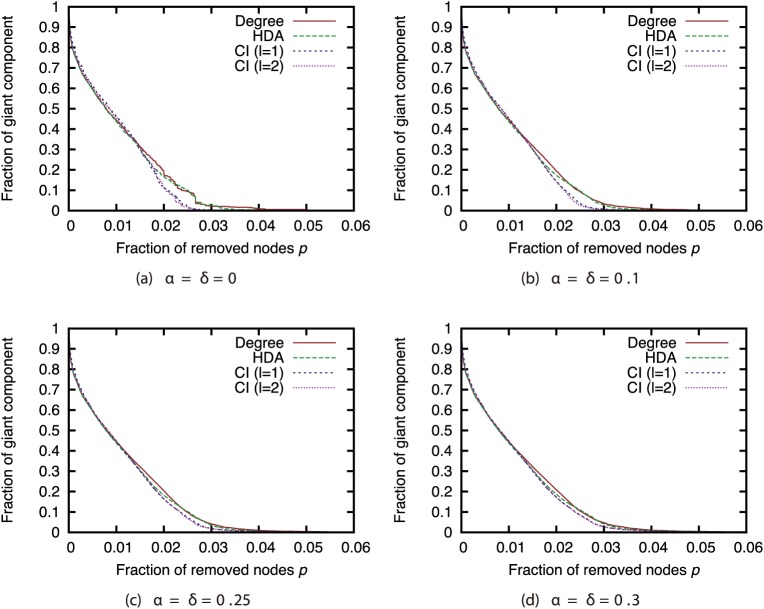
Fraction of GC vs. fraction *p* of removed nodes: Comparison of attack strategies (network: AS).

**Fig 7:**
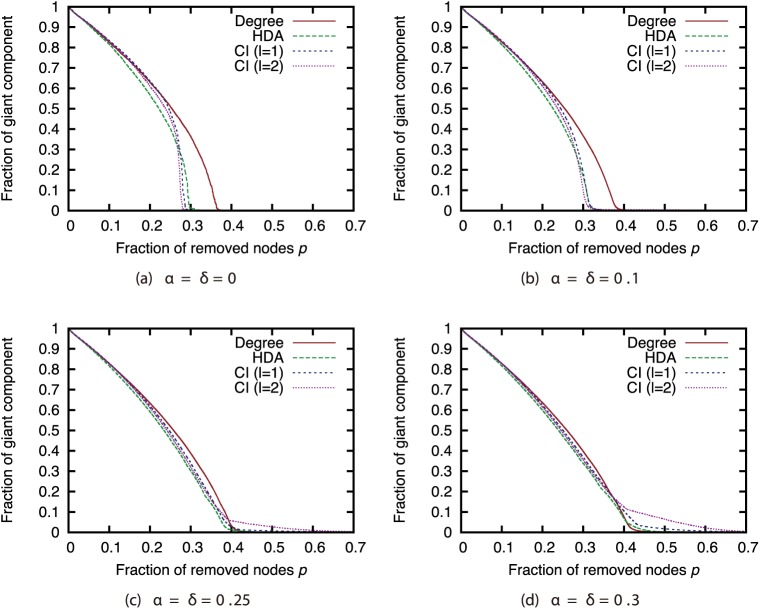
Fraction of GC vs. fraction *p* of removed nodes: Comparison of attack strategies (network: P2P).

**Fig 8:**
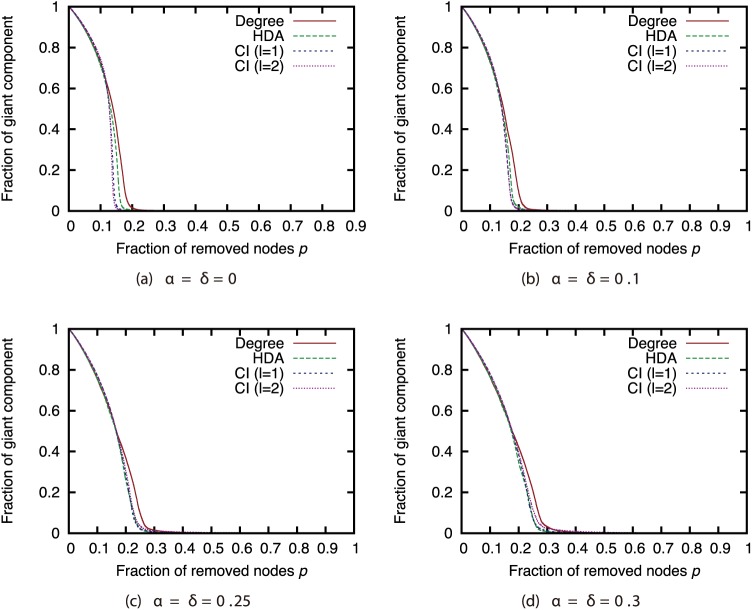
Fraction of GC vs. fraction *p* of removed nodes: Comparison of attack strategies (network: BA).

**Fig 9:**
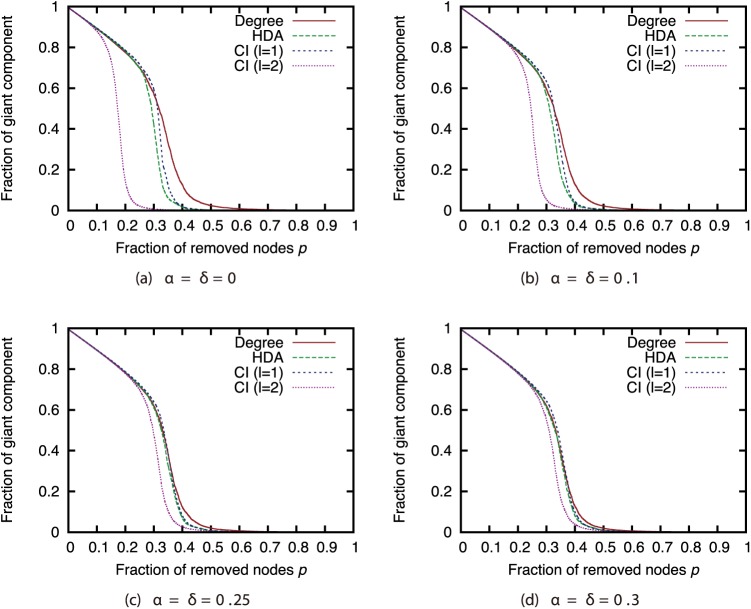
Fraction of GC vs. fraction *p* of removed nodes: Comparison of attack strategies (network: GRG).

Next, we compared the attack strategies under the same magnitudes of incompleteness (Figs 6, 7, 8 and 9). Our results show that when the magnitude of incompleteness is small, the CI strategy can decrease the size of the GC with the removal of fewer nodes than the Degree strategy. In particular, in the GRG network, CI (*l* = 2) is more effective than other strategies when the complete network is available. By contrast, when the magnitude of incompleteness is large, the attack strategies deliver similar effects. Even in the GRG network, the difference between CI and other strategies is small. These results suggest that CI, which is a state-of-the-art attack strategy, experiences a decline in effectiveness similar to that with other strategies when the magnitude of incompleteness is large.

**Fig 10:**
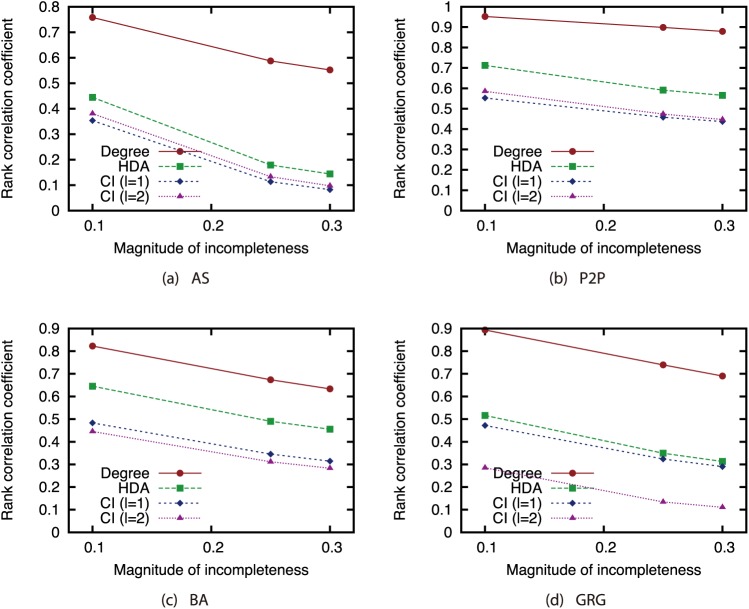
Rank correlation coefficient vs. the magnitude of incompleteness *α* and *δ*.

To investigate how the node rankings based on the attack strategies are changed by link errors, we investigated the Spearman’s rank correlation between the node ranking obtained from the ground-truth network *G* and node ranking obtained from incomplete network *G*′. Fig 10 shows the relation between the magnitude of incompleteness and the rank correlation coefficient. This result confirms that the Degree strategy is robust against link errors while the CI strategy is sensitive against link errors. Although CI is effective when the complete knowledge on the network is available, the node ranking based on CI is significantly affected by link errors. This is the cause of the decline in the effectiveness of CI under link errors.

### Effects of node sampling

**Fig 11:**
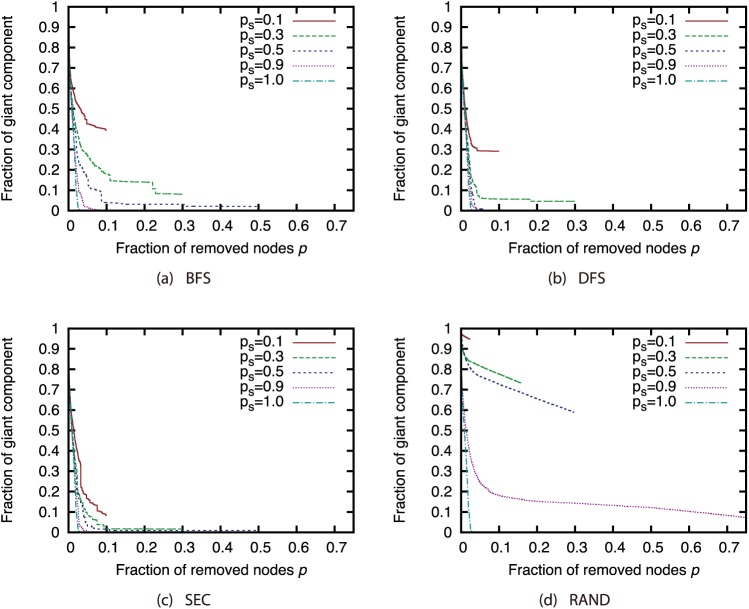
Fraction of GC vs. fraction *p* of removed nodes: Comparison of different sample sizes (network: AS; attack strategy: Degree).

**Fig 12:**
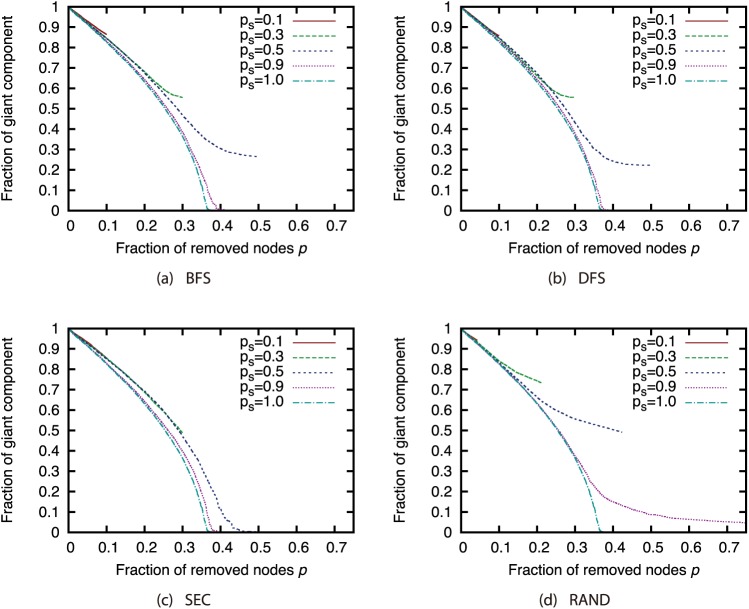
Fraction of GC vs. fraction *p* of removed nodes: Comparison of different sample sizes (network: P2P; attack strategy: Degree).

**Fig 13:**
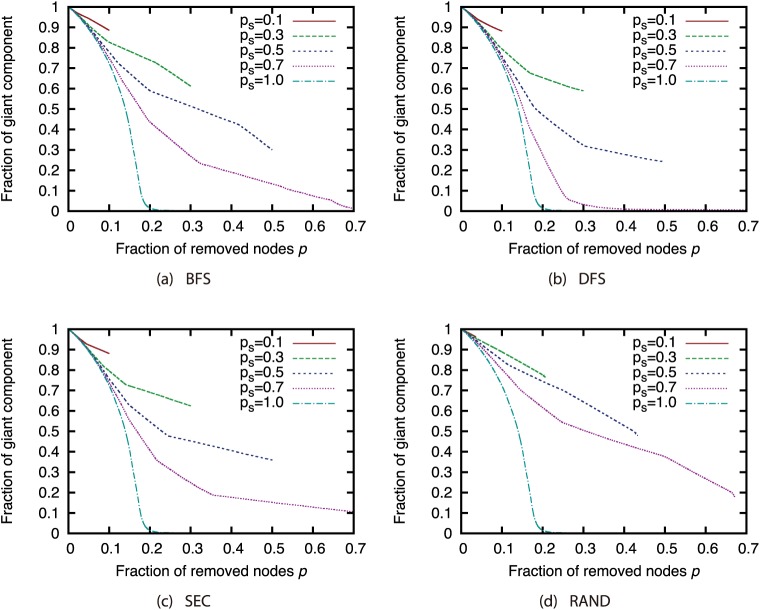
Fraction of GC vs. fraction *p* of removed nodes: Comparison of different sample sizes (network: BA; attack strategy: Degree).

**Fig 14:**
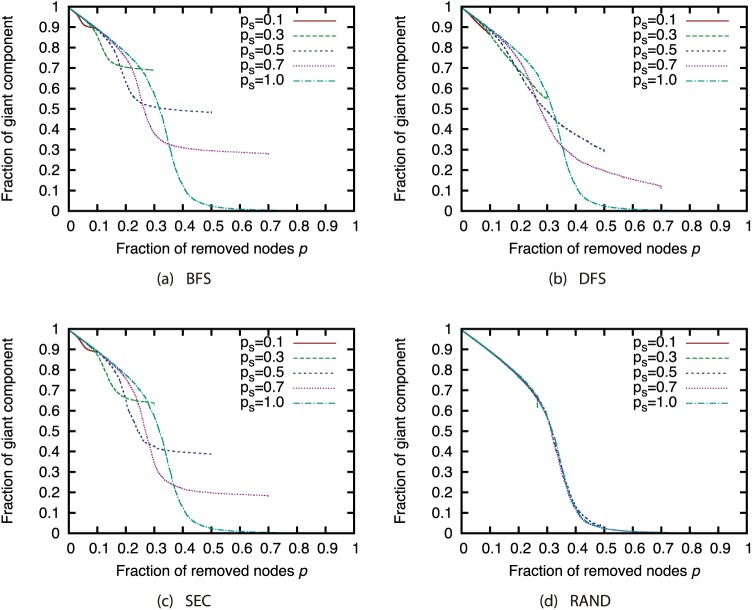
Fraction of GC vs. fraction *p* of removed nodes: Comparison of different sample sizes (network: GRG; attack strategy: Degree).

Next, we investigated how node sampling affects the effectiveness of the attack strategies. Figs 11, 12, 13 and 14 show the relationship between the proportion of removed nodes and the proportion of the GC in the AS, P2P, BA, and GRG networks under the Degree attack strategy. Here, we compared the results of different sample sizes. Note that for the experiments in this section, we did not remove a larger proportion of nodes than the sample sizes. Therefore, the graphs may contain some discontinuities.

Our results show that random sampling has an extremely strong effect on the Degree strategy in each network. However, the effects of other sampling methods are much smaller than those of random sampling. Fig 11 shows that sample sizes of 0.3 and greater under sampling methods other than random sampling have hardly any effect on the effectiveness of the Degree strategy in the AS network. In addition, Fig 12 shows that sample sizes of 0.3 and greater under the SEC method have hardly any effect on the effectiveness of the Degree strategy in the P2P network. In BA and GRG networks, although BFS, DFS, and SEC have considerable effects on the degree strategy, the effects are smaller than the effects of random sampling. These results suggest that the effectiveness of the attack strategies under sampling methods other than random sampling do not decline substantially, even under a certain degree of incompleteness of node information. It is commonly known that high-degree nodes are easy to sample when using BFS, DFS, and SEC [17]. Therefore, the effectiveness of the attack strategies should not decline much in sampled networks, which include many nodes susceptible to high-degree attacks. A closer look at the differences among BFS, DFS, and SEC reveals that SEC sampling in particular has little effect on the attack strategies. However, for sample sizes of 50% or greater, there is not much difference among the three sampling methods.

**Fig 15:**
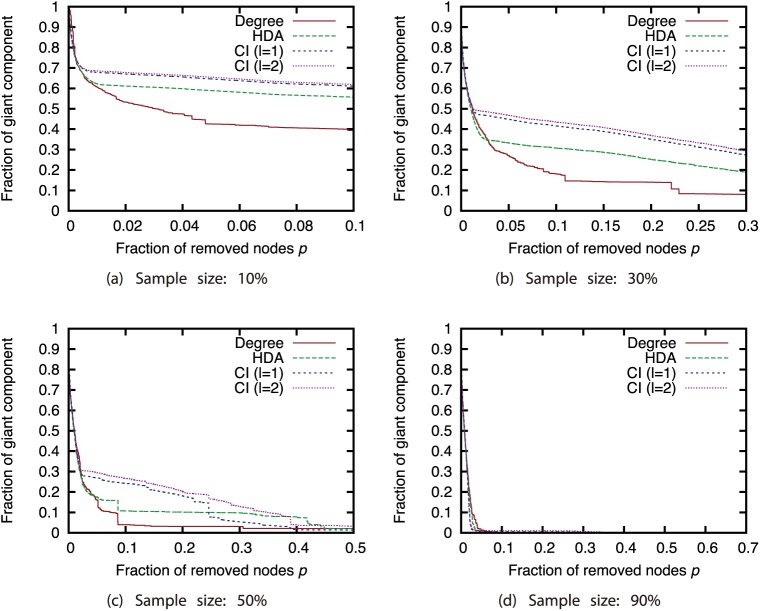
Fraction of GC vs. fraction *p* of removed nodes: Comparison of attack strategies (sampling method: BFS; network: AS).

**Fig 16:**
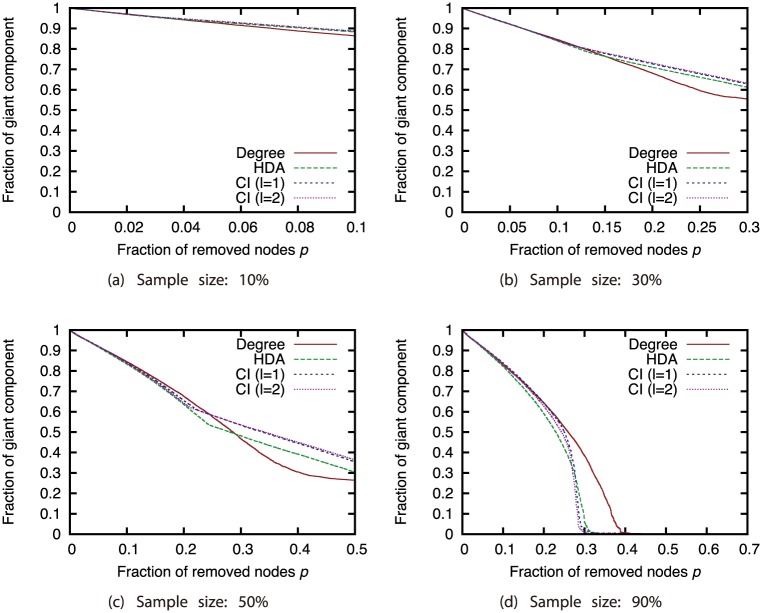
Fraction of GC vs. fraction *p* of removed nodes: Comparison of attack strategies (sampling method: BFS; network: P2P).

**Fig 17:**
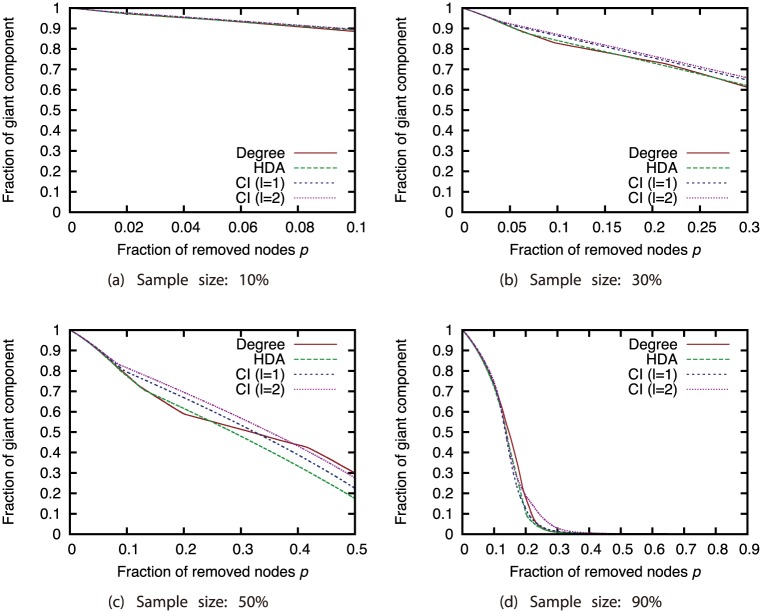
Fraction of GC vs. fraction *p* of removed nodes: Comparison of attack strategies (sampling method: BFS; network: BA).

**Fig 18:**
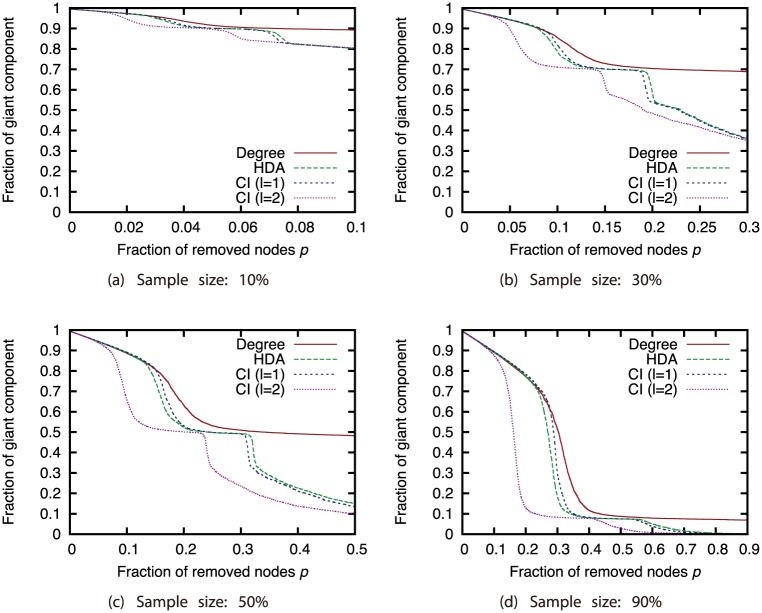
Fraction of GC vs. fraction *p* of removed nodes: Comparison of attack strategies (sampling method: BFS; network: GRG).

Next, we compared different attack strategies under the same sampling methods and sample sizes (Figs 15, 16, 17 and 18). Here, we present the results with BFS as the sampling method. The results for the other sampling methods are in Supporting Information (S1, S2, S3, S4, S5, S6, S7, S8, S9, S10, S11 and S12 Figs). These results show that CI strategy is more effective than the Degree strategy when the sample size is 90%, but its effectiveness declines greatly when the sample size is 50% or less except for the GRG network.

**Fig 19:**
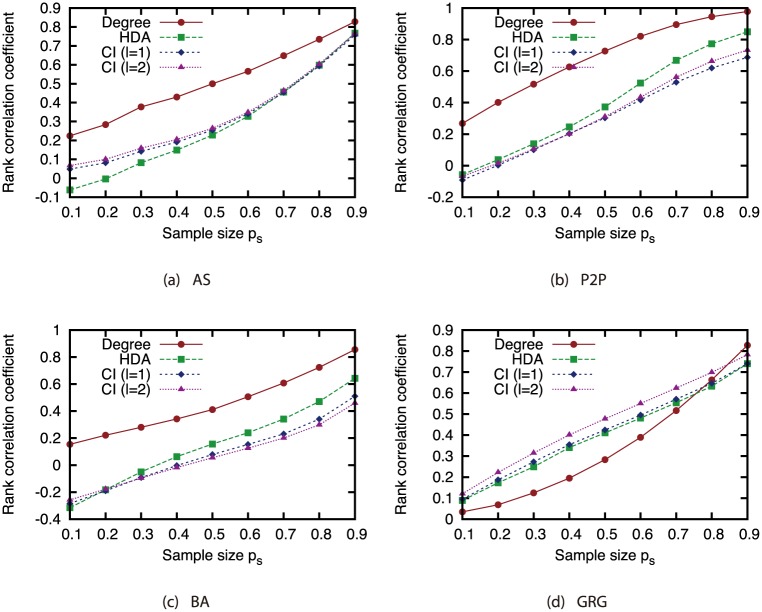
Rank correlation coefficient vs. sample size (sampling method: BFS).

We again investigated the Spearman’s rank correlation between the node ranking obtained from the ground-truth network *G* and node ranking obtained from sampled network *G*′ to investigate how the node rankings based on the attack strategies are changed by node sampling. For calculating the rank correlation, we considered that non-sampled nodes have the lowest rank. Fig 19 shows the relation between the sample size and the rank correlation coefficient. These results show that the rank correlation smoothly decreases as the sample size decreases. We can find that the degree has higher rank correlation than CI in AS, P2P, and BA networks. This confirms that the Degree strategy is robust and the CI strategy is sensitive against network sampling.

### Applications to network immunization

**Fig 20:**
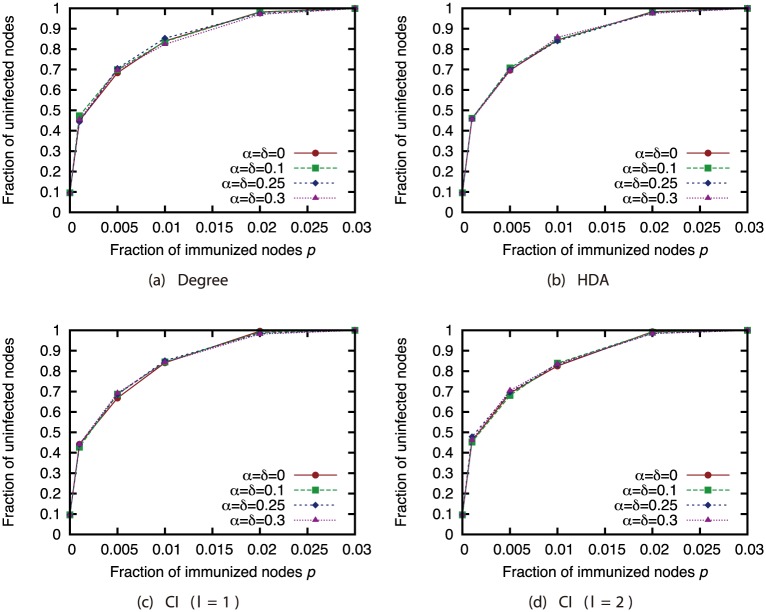
Fraction of nodes not infected by the virus at convergence of virus spread vs. fraction *p* of immunized nodes: Effects of link errors (network: AS).

**Fig 21:**
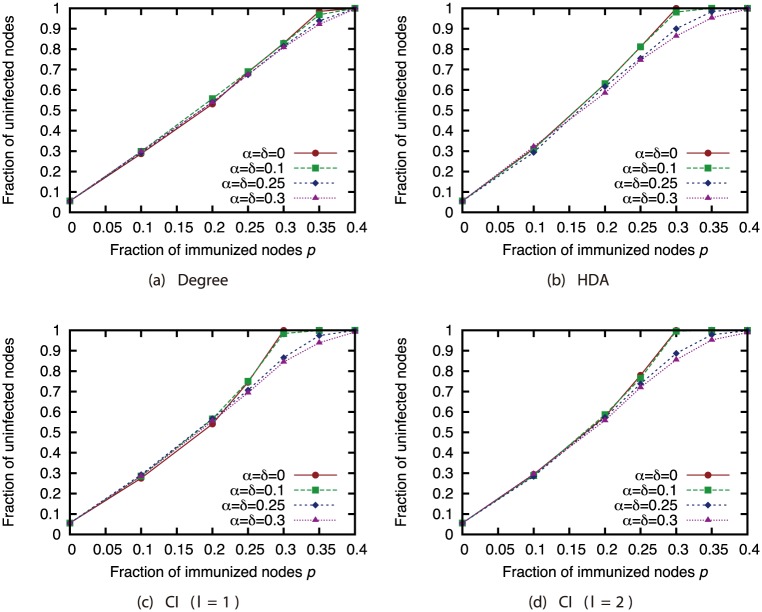
Fraction of nodes not infected by the virus at convergence of virus spread vs. fraction *p* of immunized nodes: Effects of link errors (network: P2P).

**Fig 22:**
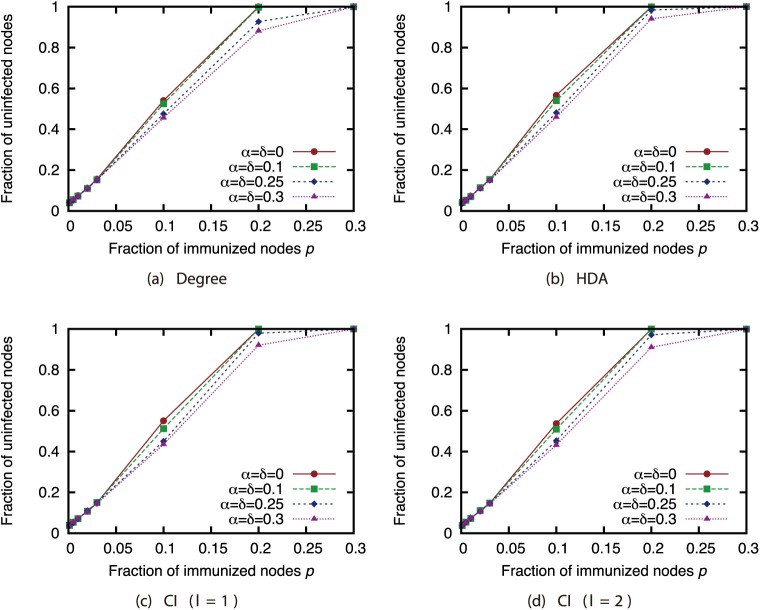
Fraction of nodes not infected by the virus at convergence of virus spread vs. fraction *p* of immunized nodes: Effects of link errors (network: BA).

**Fig 23:**
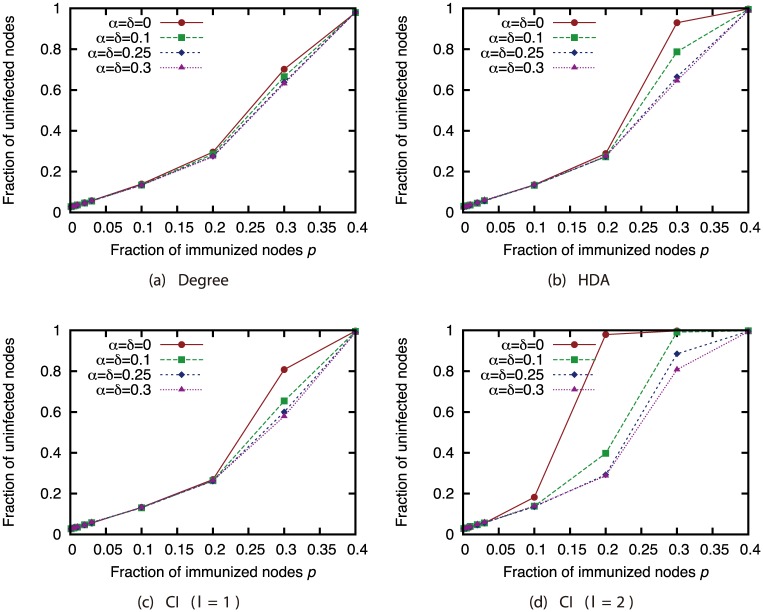
Fraction of nodes not infected by the virus at convergence of virus spread vs. fraction *p* of immunized nodes: Effects of link errors (network: GRG).

Finally, we investigated the effectiveness of the attack strategies when applied to the network immunization problem. The size of the GC in the experiments thus far expresses the extent of the damage from virus spread in the worst case. For this section, we conducted virus spread simulations based on the SIR model with immunized nodes, and we evaluated the average extent of the damage from virus spread. Figs 20, 21, 22 and 23 show the results for the network with link errors. These figures show the relationship between the proportion of immunized nodes and the proportion of nodes ultimately not infected by the virus.

These results show that in most networks, the effectiveness of each attack strategy at controlling virus spread is essentially the same, for networks with link errors as well as complete networks. Except for the results of CI (*l* = 2) in the GRG network, we confirmed that the effects of link errors are not so large.

**Fig 24:**
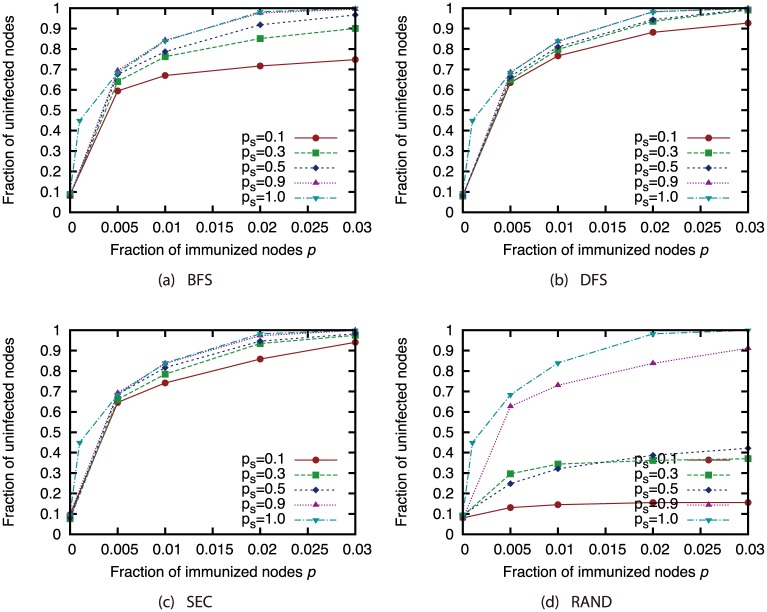
Fraction of nodes not infected by the virus at convergence of virus spread vs. fraction *p* of immunized nodes: Effects of network sampling (immunization strategy: Degree; network: AS).

**Fig 25:**
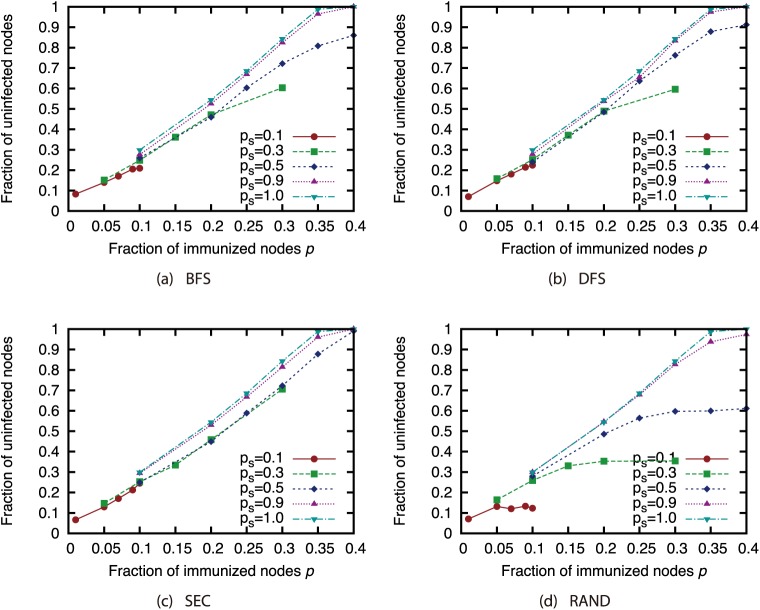
Fraction of nodes not infected by the virus at convergence of virus spread vs. fraction *p* of immunized nodes: Effects of network sampling (immunization strategy: Degree; network: P2P).

**Fig 26:**
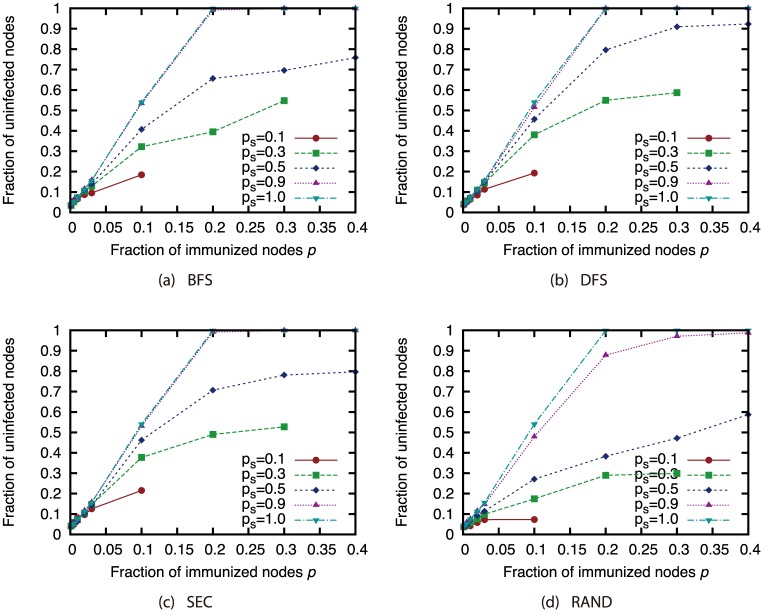
Fraction of nodes not infected by the virus at convergence of virus spread vs. fraction *p* of immunized nodes: Effects of network sampling (immunization strategy: Degree; network: BA).

**Fig 27:**
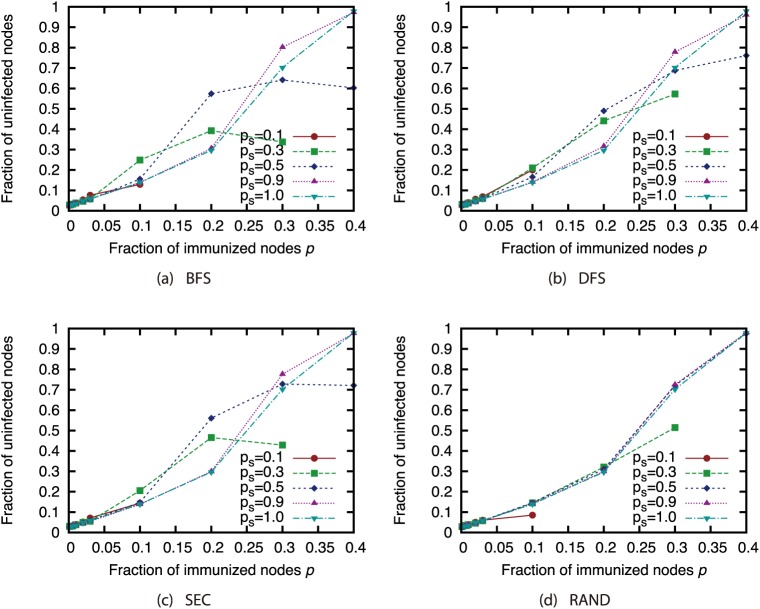
Fraction of nodes not infected by the virus at convergence of virus spread vs. fraction *p* of immunized nodes: Effects of network sampling (immunization strategy: Degree; network: GRG).

Next, we present the results when we used the sampled network to determine which nodes to immunize. Figs 24, 25, 26 and 27 show the results for the AS, P2P, BA and GRG networks. Here, we used the Degree attack strategy. Fig 24 shows that the virus spread was controlled to a certain extent even with the extremely small sample size of 10% under sampling methods other than random sampling in the AS network, which features a heavily skewed degree distribution. For other networks, the effects of sampling methods other than random sampling are not so severe when the sample size is 50% or higher. In the GRG network, using the complete network does not always achieve the highest fraction of unifected nodes. This is due to the fact that the Degree strategy is not the best strategy in the GRG network. Using the sampled networks sometimes achieves better selection of immunized nodes than using the complete networks.

These results suggest that the effects of link errors and sampling are not so severe when the attack strategies are applied to immunization. As shown in the previous sections, node rankings used in the network attack strategies are significantly affected by the network incompleteness, and the size of the GC when the attack strategies are applied is also affected. Compared to these effects, the effects of network incompleteness in the immunization problem is considered to be not severe. However, proper attention must be paid when networks are obtained under random sampling or when sample sizes are extremely small.

## Conclusion

In this paper, we have investigated the effects of link errors and network sampling on the effectiveness of network attack strategies. We have performed extensive simulations using real network data and network generation models. For the effects of link errors, we validated previous findings [5]. Namely, we showed that in networks with highly skewed degree distributions in particular, network attack strategies are robust against link errors. We have also shown that the effectiveness of network attack strategies is much degraded when the network is obtained from random sampling, whereas the detrimental effects of network sampling on network attack strategies are small when using biased sampling strategies such as BFS, DFS, and SEC. However, when the sample size is quite small (i.e., less than 30%), the effects of network sampling are not negligible. Comparing the different attack strategies, we have found that the state-of-the-art CI strategy is not robust against network uncertainty, and when the level of network uncertainty is large, the effectiveness of CI is comparable with that of the conventional Degree strategy.

Moreover, we have examined the effectiveness of network attack strategies when applied to the network immunization problem. Overall, the detrimental effects of link errors and network sampling are not severe for the network immunization problem. Our results show that the effects of link errors on the effectiveness of immunization are small. Although the effects of random sampling are large, those of other sampling methods are small. Particularly for the AS network, the effectiveness of immunization based on Degree using only a 10% sample of the network obtained with SEC and DFS is comparable with that using the complete network.

We recognize that there are some limitations of this study, and these suggest future research directions. First, how combinations of different types of incompleteness affect the network attack strategies is still unclear. In reality, sampled networks also include link errors. It is therefore important to investigate the extent to which these different types of error in a network affect the network attack strategies. Second, the generalizability of the results in this study should be validated. In particular, the relation between the topological characteristics of the ground-truth network and the robustness of the network attack strategies should be investigated in the future research. Moreover, new attack strategies [2, 4] have been proposed since CI, and analyzing the robustness of these strategies is also important future work. Third, to understand the effectiveness of network attack strategies for the network immunization problem, more detailed investigations are still necessary. In this paper, we only consider the scenario where the virus spreading process is initiated from a randomly selected node. Other scenarios such as where multiple nodes are infected or high-degree nodes are infected should be also investigated. Moreover, to investigate the effectiveness of the strategies under different infection and recovery rates and under different spreading models such as the SIS and the SEIR models is also interesting.

## Supporting information

S1 Fig Fraction of GC vs. fraction *p* of removed nodes: Comparison of attack strategies (sampling method: DFS; network: AS). (EPS)

S2 Fig Fraction of GC vs. fraction *p* of removed nodes: Comparison of attack strategies (sampling method: DFS; network: P2P). (EPS)

S3 Fig Fraction of GC vs. fraction *p* of removed nodes: Comparison of attack strategies (sampling method: DFS; network: BA). (EPS)

S4 Fig Fraction of GC vs. fraction *p* of removed nodes: Comparison of attack strategies (sampling method: DFS; network: GRG). (EPS)

S5 Fig Fraction of GC vs. fraction *p* of removed nodes: Comparison of attack strategies (sampling method: SEC; network: AS). (EPS)

S6 Fig Fraction of GC vs. fraction *p* of removed nodes: Comparison of attack strategies (sampling method: SEC; network: P2P). (EPS)

S7 Fig Fraction of GC vs. fraction *p* of removed nodes: Comparison of attack strategies (sampling method: SEC; network: BA). (EPS)

S8 Fig Fraction of GC vs. fraction *p* of removed nodes: Comparison of attack strategies (sampling method: SEC; network: GRG). (EPS)

S9 Fig Fraction of GC vs. fraction *p* of removed nodes: Comparison of attack strategies (sampling method: RAND; network: AS). (EPS)

S10 Fig Fraction of GC vs. fraction *p* of removed nodes: Comparison of attack strategies (sampling method: RAND; network: P2P). (EPS)

S11 Fig Fraction of GC vs. fraction *p* of removed nodes: Comparison of attack strategies (sampling method: RAND; network: BA). (EPS)

S12 Fig Fraction of GC vs. fraction *p* of removed nodes: Comparison of attack strategies (sampling method: RAND; network: GRG). (EPS)
